# APOE-ε4-related differences in left thalamic microstructure in cognitively healthy adults

**DOI:** 10.1038/s41598-020-75992-9

**Published:** 2020-11-13

**Authors:** Jilu P. Mole, Fabrizio Fasano, John Evans, Rebecca Sims, Emma Kidd, John P. Aggleton, Claudia Metzler-Baddeley

**Affiliations:** 1 Cardiff University Brain Research Imaging Centre (CUBRIC), School of Psychology, Cardiff University, Maindy Road, Cathays, Cardiff, CF24 4HQ UK; 2 Siemens Healthcare, Henkestrasse 127, 91052 Erlangen, Germany; 3 Division of Psychological Medicine and Clinical Neuroscience, School of Medicine, Cardiff University, Haydn Ellis Building, Maindy Road, Cathays, Cardiff, CF24 4HQ UK; 4 School of Pharmacy and Pharmaceutical Sciences, Cardiff University, Redwood Building, King Edward VII Avenue,, Cardiff, CF10 3NB UK

**Keywords:** Cognitive ageing, Myelin biology and repair, Neural ageing

## Abstract

*APOE*-ε4 is a main genetic risk factor for developing late onset Alzheimer’s disease (LOAD) and is thought to interact adversely with other risk factors on the brain. However, evidence regarding the impact of *APOE*-ε4 on grey matter structure in asymptomatic individuals remains mixed. Much attention has been devoted to characterising *APOE*-ε4-related changes in the hippocampus, but LOAD pathology is known to spread through the whole of the Papez circuit including the limbic thalamus. Here, we tested the impact of *APOE*-ε4 and two other risk factors, a family history of dementia and obesity, on grey matter macro- and microstructure across the whole brain in 165 asymptomatic individuals (38–71 years). Microstructural properties of apparent neurite density and dispersion, free water, myelin and cell metabolism were assessed with Neurite Orientation Density and Dispersion (NODDI) and quantitative magnetization transfer (qMT) imaging. *APOE*-ε4 carriers relative to non-carriers had a lower macromolecular proton fraction (MPF) in the left thalamus. No risk effects were present for cortical thickness, subcortical volume, or NODDI indices. Reduced thalamic MPF may reflect inflammation-related tissue swelling and/or myelin loss in *APOE*-ε4. Future prospective studies should investigate the sensitivity and specificity of qMT-based MPF as a non-invasive biomarker for LOAD risk.

## Introduction

As the global population ages, an increasing number of people over 65 will develop dementia due to late onset Alzheimer’s disease (LOAD)^1^. LOAD is characterized by the development of amyloid-β plaques and neurofibrillary tau tangles that spread from limbic regions to neocortical areas^2–4^. As these pathological processes are thought to accumulate over many years^5^, it may be possible to identify brain changes related to heightened risk in asymptomatic individuals prior to the onset of memory impairment.

Carriage of the Apolipoprotein E (*APOE)*-ε4 genotype is the best-established genetic risk factor of LOAD^6,7^. *APOE* is the main cholesterol carrier in the brain that supports lipid transport, myelination, synaptic repair and the regulation of amyloid-β aggregation and clearance^8^. Individuals who carry the *APOE*-ε4 isoform compared to those with *APOE*-ε2 and -ε3 show an earlier onset of LOAD^6,9^ and a larger burden of amyloid-β plaques^10–14^. Such harmful effects of *APOE*-ε4 are heightened in individuals with a family history of LOAD^15,16^, probably due to the presence of other polygenic risk variants such as those of *TREM2*^17,18^. In addition, *APOE*-ε4 is known to combine adversely with lifestyle-related risk notably central obesity^19,20^. Excessive abdominal visceral fat can lead to the metabolic syndrome, type 2 diabetes, and cardiovascular disease^21^ and obese *APOE*-ε4 carriers are more likely to develop hypertension, inflammation and insulin resistance^22,23^.

Much attention has been devoted to characterizing *APOE*-ε4-related changes in medial temporal lobe regions, notably in the hippocampus and parahippocampal regions^24–26^ due to their importance for episodic memory. Hippocampal volume loss on magnetic resonance imaging (MRI) is also one of the diagnostic biomarkers of LOAD^27^. However, hippocampal atrophy is lacking in specificity^28^ and usually occurs in more advanced disease stages^29^. Indeed, evidence regarding hippocampal atrophy in *APOE*-ε4 carriers is mixed and is often thought to result from the inclusion of older participants with underlying LOAD pathology^30,31^. It, therefore, stands to reason that hippocampal volume loss may not be sufficiently sensitive to detect very early disease changes and it has been proposed that focusing on specific hippocampal subregions such as CA1 and subiculum may be more promising^32,33^. However, it is also possible that limbic regions other than the hippocampus may play an important role in the development of LOAD. Notably, it has been recognised for a while that LOAD pathology may spread through the whole of the Papez circuit and may critically involve the limbic thalamus^4^. For instance, neurofibrillary accumulations in the anterodorsal thalamic nucleus have been found at the same time as those in the hippocampus in LOAD brains^34^ and reduced thalamic MRI volume has been observed in amnestic Mild Cognitive Impairment (MCI)^35^, LOAD^36^ and presymptomatic presenilin 1 mutation carriers^37^. Similarly, Positron Emission Tomography (PET) studies have found *APOE-*ε4 state to accelerate longitudinal reductions in glucose metabolism in the thalamus and frontal, parietal, and posterior cingulate regions in MCI^38^. Reduced glucose metabolism in anterior and posterior cingulate cortices, retrosplenial, precuneus, parietal cortex, hippocampus and thalamus was also observed in cognitively healthy middle-aged *APOE-*ε4 carriers^39^, suggesting that metabolic tissue changes in regions beyond the hippocampus can already occur at asymptomatic stages^40^.

While PET imaging is sensitive to metabolic changes and can identify amyloid-β and tau burden^41^, it is invasive and expensive and, therefore, difficult to scale up. Recent advances in non-invasive multi-parametric quantitative MRI (qMRI) methods can reveal subtle microstructural brain changes and promise to provide alternative imaging markers that may be sensitive to early risk-related changes. Up to now qMRI measurements have primarily been studied in LOAD patients and animal models, thus evidence with regards to the effects of risk factors in asymptomatic individuals is sparse.

To address this gap in the literature, we went beyond morphological analyses by employing multi-parametric qMRI to study the effects of *APOE*-ε4, Family History (FH) of dementia and obesity on cortical and subcortical grey matter in 165 asymptomatic individuals from the Cardiff Ageing and Risk of Dementia Study (CARDS)^42–44^ (Table 1). More specifically we applied indices sensitive to neurite dispersion and density, free water, myelin and cell metabolism from Neurite Orientation Density and Dispersion Imaging (NODDI)^45^, quantitative magnetization transfer (qMT)^46–49^ and T_1_-relaxometry^50^ (Table 2).

**Table 1:** Summary of demographic, genetic, and lifestyle risk information of CARDS participants.

	Mean (SD) (range)
Sample size n	165
Age (in years)	55.7 (8.2) (38–71)
Females	57%
NART-IQ	116.8 (6.7) (96–128)
MMSE	29.1 (0.9) (27–30)
FH +	35.8%
*APOE4* +	38.8%
WHR	1.4 (0.5) (0.7–2.2)
Systolic BP (mm Hg)	132 (18.8) (68.3–196)
Diastolic BP (mm Hg)	83.3 (9.4) (58.7–118.7)
Smokers	5.5%
Diabetes	1.8%
Alcohol units per week	7.4 (9.4) (0–60)
PHQ-9 Depression score	2.6 (2.9) (0–13)

*APOE* = Apolipoprotein-E based on DNA extraction and *APOE* genotyping of saliva samples using TaqMan genotyping of single nucleotide polymorphism (SNP) rs7412 and KASP genotyping of SNP rs429358. FH = Family History of a first degree relative affected by Alzheimer’s or Lewy body disease or vascular dementia. MMSE = Mini Mental State Exam (maximum score = 30)^42^, NART-IQ = National Adult Reading Test- Intelligence Quotient^66^, PHQ-9 = Patient Health Questionnaire (maximum score = 27)^109^. WHR = Waist-to-Hip-Ratio.

**Table 2:** Overview of the quantitative microstructural indices and their interpretation in grey matter.

MRI modality	Index	Apparent grey matter property	Hypothesised changes with LOAD risk
Diffusion NODDI	ICSF	Neurite density	Increases with tau pathology^55^/Reduction in MCI and AD patients^52–54^
	ODI	Neurite dispersion	Increase/Reduction
	ISOSF	Free water	Increase
qMT	MPF	Macromolecules (e.g. myelin)	Reduction
	*k*_*f*_	Mitochondrial metabolism	Increase in acute inflammation^83^; Reduction in low-level inflammation^125^ and in MCI and AD patients^59–61^
Relaxometry	R_1_	free water, myelin, iron	Increase/Reduction^62^

*AD* Alzheimer's disease, *ICSF* intracellular signal fraction, *ISOSF* isotropic signal fraction, *k*_*f*_ forward exchange rate, *MCI* mild cognitive impairment, *MPF* macromolecular proton fraction, *NODDI* neurite orientation dispersion and density imaging, *ODI* orientation dispersion index, *qMT* quantitative magnetization transfer.

NODDI fits a three-compartment biophysical tissue model to diffusion-weighted data acquired with a two-shell (b-values of 1200 s/mm^2^ and 2400 s/mm^2^) High Angular Resolution Diffusion Imaging (HARDI)^51^ protocol to separate isotropic from intra- and extracellular diffusion compartments^45^. This allows the calculation of the isotropic signal fraction (ISOSF), an estimate of free water, and the intracellular signal fraction (ICSF), i.e. the fraction of the tissue comprised of neurites. In addition, NODDI yields the orientation dispersion index (ODI) that reflects the spatial configuration of neurite structures (Table 2). Recent studies reported ICSF and ODI reductions in grey and white matter of patients with MCI, LOAD and young onset AD^52–54^. For instance, Fu et al. (2019) found decreased ICSF and ODI in the corpus callosum in MCI and LOAD patients, while Colgan et al.^55^ reported positive correlations between ICSF and histological measurements of hyperphosphorylated tau protein in the hippocampus of rTg4510 mice.

The qMT method models the exchange rate between macromolecular protons and protons in surrounding free water when macromolecular protons are selectively saturated by a radiofrequency pulse with a frequency that is off-resonance for protons in free water^46–49^. This allows the quantification of a number of parameters including the macromolecular proton fraction (MPF) and the magnetization transfer exchange rate *k*_*f*_^49^. In combined neuroimaging and histology studies of Shiverer mice and puppies^56–58^, MPF has been shown to be highly sensitive to the myelin content in white matter such that MPF increases with the amount of myelin. MPF in the anterior hippocampus was also found to distinguish healthy controls from MCI and LOAD patients^59^. Furthermore, MCI and LOAD patients exhibit a reduced rate of magnetization transfer *k*_*f*_ in grey and white matter^59–61^ suggesting reduced cell metabolism^60^. Finally, indices from relaxometry imaging such as the longitudinal relaxation rate R_1_ have been proposed as non-invasive biomarkers of LOAD^62^. R_1_ values are influenced by microstructural characteristics such as tissue density, macromolecular, protein and lipid composition, and paramagnetic atoms. A number of patient and preclinical studies have reported increases in R_1_ that may reflect LOAD pathology, although the precise mechanisms underpinning these changes remain unknown (see for review^62^)**.**

Here, we characterised age and risk-related differences in mean values of ICSF, ISOSF, ODI, MPF, *k*_*f*_and R_1_ across cortical and subcortical grey matter regions that were segmented from T_1_—weighted images with the FreeSurfer image analysis suite (version 5.3)^63^. Microstructural changes were compared with differences in standard morphological metrics of cortical thickness and subcortical volumes. We expected to see risk effects in brain regions known to be early affected in LOAD including limbic regions of the hippocampus, parahippocampus, entorhinal cortex, posterior cingulate cortex as well as thalamus^2,4,34,64^. We hypothesised that *APOE*-ε4, a positive FH, and central obesity [measured with the Waist-Hip-Ratio (WHR)] would be associated with reduced ICSF, R_1_, MPF and *k*_*f*_ as well as with increased ISOSF and ODI but with no differences in cortical thickness and/or subcortical volume. In addition, we expected to see the largest differences in those individuals at greatest risk, i.e. in obese *APOE*-ε4 carriers with a positive FH.

## Results

Microstructural and morphological dependent variables were fitted to a general linear model in SPSS version 26^65^. All data were examined for outliers defined as above or below three times of the interquartile range (75th percentile value–25th percentile value). This led to an exclusion of 0.6% of the microstructural but no exclusions of the morphological data.

Separate multivariate analyses of covariance (MANCOVA) were carried out to test for the effects of *APOE* genotype (ε4 + , ε4-), FH (FH + , FH-) and WHR (WHR + , WHR-) on brain morphology (cortical thickness and subcortical volume measures) and on each of the microstructural indices (MPF, *k*_*f*_, R_1_, ISOSF, ICSF, ODI) across 68 cortical and 14 subcortical regions of interest, whilst controlling for age, sex, and IQ estimates from the revised National Adult Reading Test (NART-R)^66^. Significant omnibus effects were further investigated with post-hoc comparisons across all outcome measures. All first and post-hoc models were corrected for multiple comparisons with a False Discovery Rate (FDR) of 5% using the Benjamini–Hochberg procedure^67^ (p_BHadj_). As the aim of the study was to explore microstructural indices that could potentially provide novel biomarkers of dementia risk in future studies, a false positive rate of below 5% was regarded as an acceptable threshold to control for false positives while minimising the risk of missing any true risk-related microstructural differences. Information about effects sizes was provided with the partial eta squared index η_p_^2^ for MANCOVA analyses, Cohen’s d_z_ for group comparisons and Pearson’s r for correlational analyses.

### MANCOVAs of microstructural qMT metrics

#### MPF omnibus effects

There were main effects of sex [F(78,46) = 2.2, p_BHadj_ = 0.015, η_p_^2^ = 0.8] and of *APOE* genotype [F(78,46) = 2.6, p_BHadj_ < 0.001, η_p_^2^ = 0.8] but not of FH (p_BHadj_ = 0.137), WHR (p_BHadj_ = 0.348), age (p_BHadj_ = 0.385) or NART-IQ (p_BHadj_ = 0.497). There were no interaction effects between *APOE* and FH (p_BHadj_ = 1.000), *APOE* and WHR (p_BHadj_ = 0.974), FH and WHR (p_BHadj_ = 1.000) or *APOE*, FH and WHR (p_BHadj_ = 0.935).

#### MPF post-hoc effects

**Figure 1:**
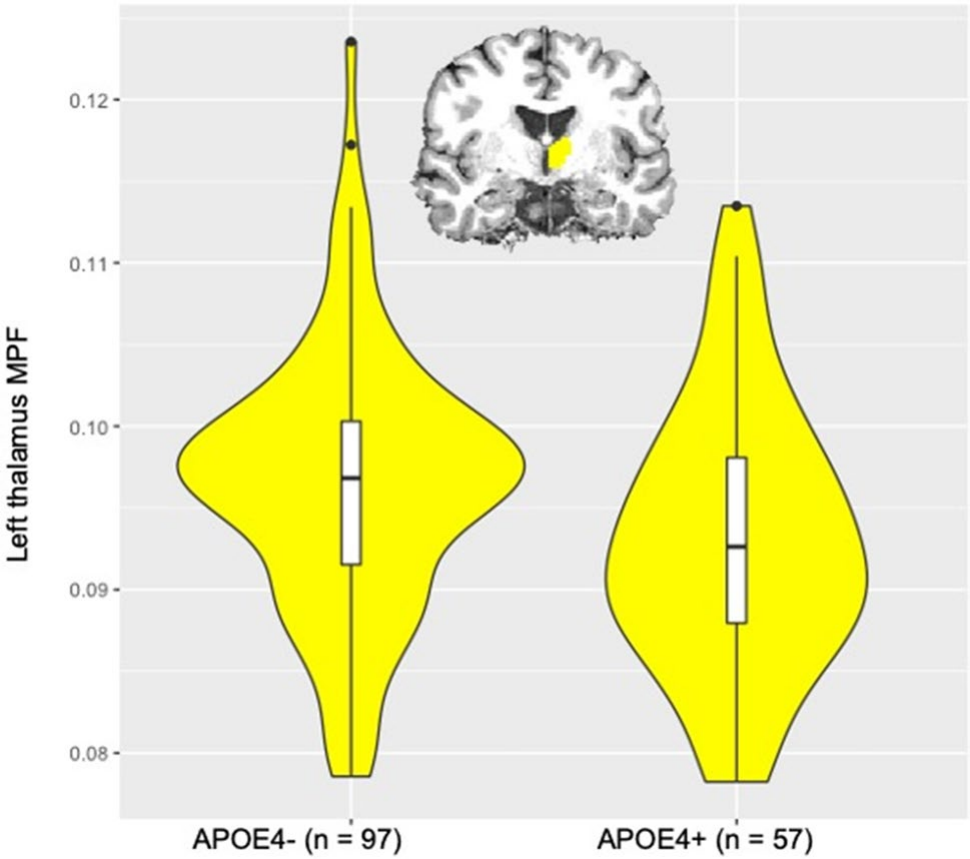
Violin plots with overlaid box plots of the difference in the macromolecular proton fraction (MPF) in the left thalamus between *APOE*-ε4 carriers (n = 57) and non-carriers (n = 97) (p_BHadj_ = 0.026). Boxplots display the median and the interquartile range and violin plots the kernel probability density, i.e. the width of the yellow area represents the proportion of the data located there.

**Figure 2:**
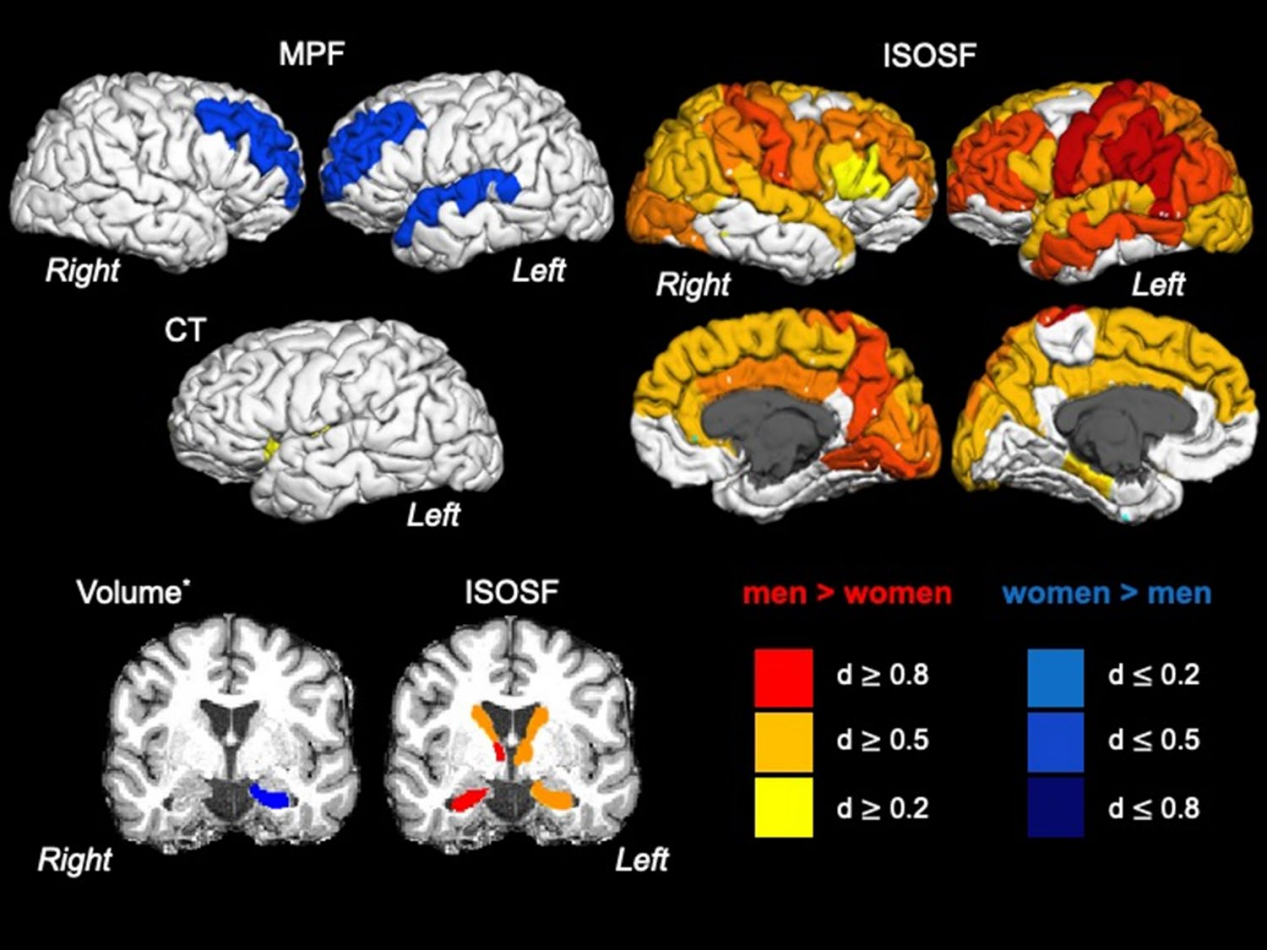
displays the effects of sex on cortical thickness (CT), subcortical volume (corrected for intracranial volume), isotropic signal fraction (ISOSF) and macromolecular proton fraction (MPF) across 34 cortical regions per hemisphere parcellated with the Desikan–Killiany atlas^121^ and seven subcortical regions per hemisphere (hippocampus, amygdala, thalamus, caudate, putamen, globus pallidus, nucleus accumbens). Region of interest segmentations were performed with FreeSurfer (version 5.3). Regions are colour-coded according to effect sizes indicated by Cohen’s d^126^. Warm colours indicate positive and blue colours negative correlations. L = Left, R = Right.

*APOE*-ε4 carriers relative to non-carriers had lower MPF in the left thalamus (Table 3) (Fig. 1). Women had higher MPF than men in the left and right rostral middle frontal cortices, in the left superior temporal cortex and the right transverse temporal cortex (Table 3) (Fig. 2).

**Table 3:** Post-hoc effects of *APOE* genotype and sex on the macromolecular proton fraction (MPF).

Effect	Side	ROI	F_(1,123)_-value	p_BHadj_
APOE	Left	Accumbens	3.985	0.214
		Amygdala	0.171	0.869
		Caudate	6.710	0.090
		Hippocampus	5.327	0.143
		Pallidum	0.099	0.891
		Putamen	1.416	0.511
		**Thalamus**	**10.772**	**0.026**
	Right	Accumbens	0.310	0.790
		Amygdala	0.125	0.868
		Caudate	3.433	0.264
		Hippocampus	6.700	0.095
		Pallidum	0.039	0.919
		Putamen	1.226	0.561
		Thalamus	5.233	0.144
	Left	Banks of superior temporal sulcus	3.424	0.261
		Caudal anterior cingulate	1.518	0.483
		Cuneus	0.631	0.689
		Entorhinal	0.002	0.986
		Frontal pole	2.579	0.320
		Fusiform	0.771	0.669
		Inferior parietal	0.886	0.631
		Inferior temporal	0.942	0.635
		Insula	6.754	0.097
		Lateral occipital	0.307	0.788
		Lateral orbito frontal	0.355	0.777
		Lingual	0.641	0.690
		Medial orbito frontal	0.001	0.993
		Middle temporal	2.653	0.318
		Paracentral	0.035	0.924
		Parahippocampal	0.150	0.865
		Pars opercularis	8.341	0.097
		Pars orbitalis	0.028	0.932
		Pars triangularis	0.019	0.945
		Postcentral	2.459	0.331
		Posterior cingulate	1.065	0.592
		Precentral	3.040	0.297
		Precuneus	0.000	0.997
		Rostral anterior cingulate	0.531	0.714
		Rostral middle frontal	0.112	0.880
		Superior frontal	0.515	0.719
		Superior parietal	0.222	0.836
		Superior temporal	1.096	0.594
		Supramarginal	2.657	0.312
		Temporal pole	3.597	0.252
		Transverse temporal	5.752	0.117
	Right	Banks of superior temporal sulcus	0.085	0.892
		Caudal anterior cingulate	6.693	0.100
		Cuneus	0.077	0.897
		Entorhinal	0.088	0.892
		Frontal pole	0.070	0.882
		Fusiform	2.047	0.416
		Inferior parietal	0.736	0.673
		Inferior temporal	0.162	0.865
		Insula	4.235	0.198
		Isthmus cingulate	0.927	0.635
		Lateral occipital	0.072	0.891
		Lateral orbito frontal	0.785	0.668
		Lingual	3.499	0.262
		Medial orbito frontal	1.979	0.407
		Middle temporal	0.130	0.876
		Paracentral	0.071	0.887
		Parahippocampal	1.994	0.409
		Pars opercularis	1.551	0.493
		Pars orbitalis	0.511	0.714
		Pars triangularis	0.001	0.986
		Pericalcerine	0.875	0.629
		Postcentral	0.074	0.895
		Posterior cingulate	1.341	0.532
		Precentral	0.303	0.784
		Precuneus	0.198	0.854
		Rostral anterior cingulate	1.850	0.429
		Rostral middle frontal	0.151	0.858
		Superior frontal	0.026	0.932
		Superior parietal	1.548	0.488
		Superior temporal	1.148	0.579
		Supramarginal	0.167	0.866
		Temporal pole	0.764	0.665
		Transverse temporal	0.155	0.867
Sex	Left	Accumbens	0.353	0.784
		Amygdala	0.014	0.956
		Caudate	1.918	0.418
		Hippocampus	0.684	0.673
		Pallidum	1.079	0.594
		Putamen	2.12	0.405
		Thalamus	2.668	0.321
	Right	Accumbens	0.126	0.874
		Amygdala	0.000	0.993
		Caudate	0.046	0.912
		Hippocampus	0.223	0.842
		Pallidum	0.697	0.673
		Putamen	2.678	0.324
		Thalamus	0.571	0.710
	Left	Banks of superior temporal sulcus	0.559	0.711
		Caudal anterior cingulate	0.459	0.742
		Cuneus	7.712	0.093
		Entorhinal	5.902	0.115
		Frontal pole	4.243	0.204
		Fusiform	0.007	0.971
		Inferior parietal	6.242	0.104
		Inferior temporal	0.191	0.854
		Insula	1.298	0.541
		Lateral occipital	0.063	0.888
		Lateral orbito frontal	0.002	0.992
		Lingual	3.095	0.293
		Medial orbito frontal	2.921	0.298
		Middle temporal	2.496	0.331
		Paracentral	0.009	0.968
		Parahippocampal	7.180	0.104
		Pars opercularis	1.169	0.578
		Pars orbitalis	1.524	0.488
		Pars triangularis	7.929	0.085
		Postcentral	0.903	0.638
		**Posterior cingulate**	**15.379**	** < 0.001**
		Precentral	0.726	0.664
		Precuneus	4.327	0.201
		Rostral anterior cingulate	0.727	0.669
		**Rostral middle frontal**	**18.725**	** < 0.001**
		Superior frontal	4.349	0.202
		Superior parietal	1.629	0.474
		**Superior temporal**	**13.584**	** < 0.001**
		Supramarginal	7.837	0.104
		Temporal pole	3.766	0.238
		Transverse temporal	7.374	0.096
	Right	BANKS of superior temporal sulcus	2.881	0.292
		Caudal anterior cingulate	4.038	0.215
		Cuneus	7.177	0.089
		Entorhinal	2.004	0.413
		Frontal pole	4.610	0.196
		Fusiform	0.097	0.886
		Inferior parietal	1.757	0.442
		Inferior temporal	0.352	0.771
		Insula	2.943	0.308
		Isthmus cingulate	0.443	0.746
		Lateral occipital	0.297	0.782
		Lateral orbito frontal	0.356	0.790
		Lingual	3.196	0.289
		Medial orbito frontal	4.570	0.195
		Middle temporal	0.360	0.793
		Paracentral	0.425	0.752
		Parahippocampal	0.975	0.625
		Pars opercularis	0.340	0.774
		Pars orbitalis	0.892	0.636
		Pars triangularis	6.046	0.106
		Pericalcerine	0.553	0.708
		Postcentral	2.934	0.301
		Posterior cingulate	1.783	0.441
		Precentral	2.025	0.415
		Precuneus	0.597	0.702
		Rostral anterior cingulate	3.205	0.282
		**Rostral middle frontal**	**11.339**	**0.031**
		Superior frontal	8.639	0.089
		Superior parietal	4.557	0.188
		Superior temporal	7.319	0.083
		Supramarginal	2.903	0.295
		Temporal pole	6.534	0.093
		**Transverse temporal**	**14.344**	** < 0.001**

p_BHadj_, 5% False Discovery Rate Benjamini–Hochberg adjusted *p* value; *ROI* region of interest. Significant results are highlighted in bold.

#### *R*_*1*_* omnibus effects*

A significant omnibus effect was only observed for *APOE* genotype [F(82,43) = 2.1, p_BHadj_ = 0.040, η_p_^2^ = 0.08]. No main effects were present for FH (p_BHadj_ = 0.215), WHR (p_BHadj_ = 0.167), age (p_BHadj_ = 0.085) sex (p_BHadj_ = 0.060) or NART-IQ (p_BHadj_ = 0.866) and no interaction effects between *APOE* and FH (p_BHadj_ = 0.256), *APOE* and WHR (p_BHadj_ = 0.582), FH and WHR (p_BHadj_ = 0.782) or *APOE*, FH and WHR (p_BHadj_ = 0.548) were observed.

#### *R*_*1*_* post-hoc effects*

No *APOE* post-hoc effects survived FDR correction (see Supplementary Table 1).

#### *k*_*f*_ omnibus *effects*

There were no significant main effects of *APOE* (p_BHadj_ = 0.813), FH (p_BHadj_ = 0.908), WHR (p_BHadj_ = 1.000), age (p_BHadj_ = 0.075), sex (p_BHadj_ = 0.975) or NART-IQ (p_BHadj_ = 0.870) and no interaction effects between *APOE* and FH (p_BHadj_ = 0.888), *APOE* and WHR (p_BHadj_ = 0.840), FH and WHR (p_BHadj_ = 0.090) or *APOE*, FH and WHR (p_BHadj_ = 0.436).

### MANCOVAs of microstructural NODDI metrics

#### ISOSF omnibus effects

There were main effects for age [F(78,42) = 2.0, p_BHadj_ = 0.03, η_p_^2^ = 0.8], sex [F(78,42) = 3.4, p_BHadj_ < 0.001, η_p_^2^ = 0.9], and NART-IQ [F(78,42) = 2.2, p_BHadj_ = 0.020, η_p_^2^ = 0.8]. No main effects were present for the risk factors of *APOE* (p_BHadj_ = 1.000), FH (p_BHadj_ = 0.060) or WHR (p_BHadj_ = 0.717) and no interaction effects between *APOE* and FH (p_BHadj_ = 0.374), *APOE* and WHR (p_BHadj_ = 0.551), FH and WHR (p_BHadj_ = 0.986) or *APOE*, FH and WHR (p_BHadj_ = 0.678) were observed.

#### ISOSF post-hoc effects

**Figure 3:**
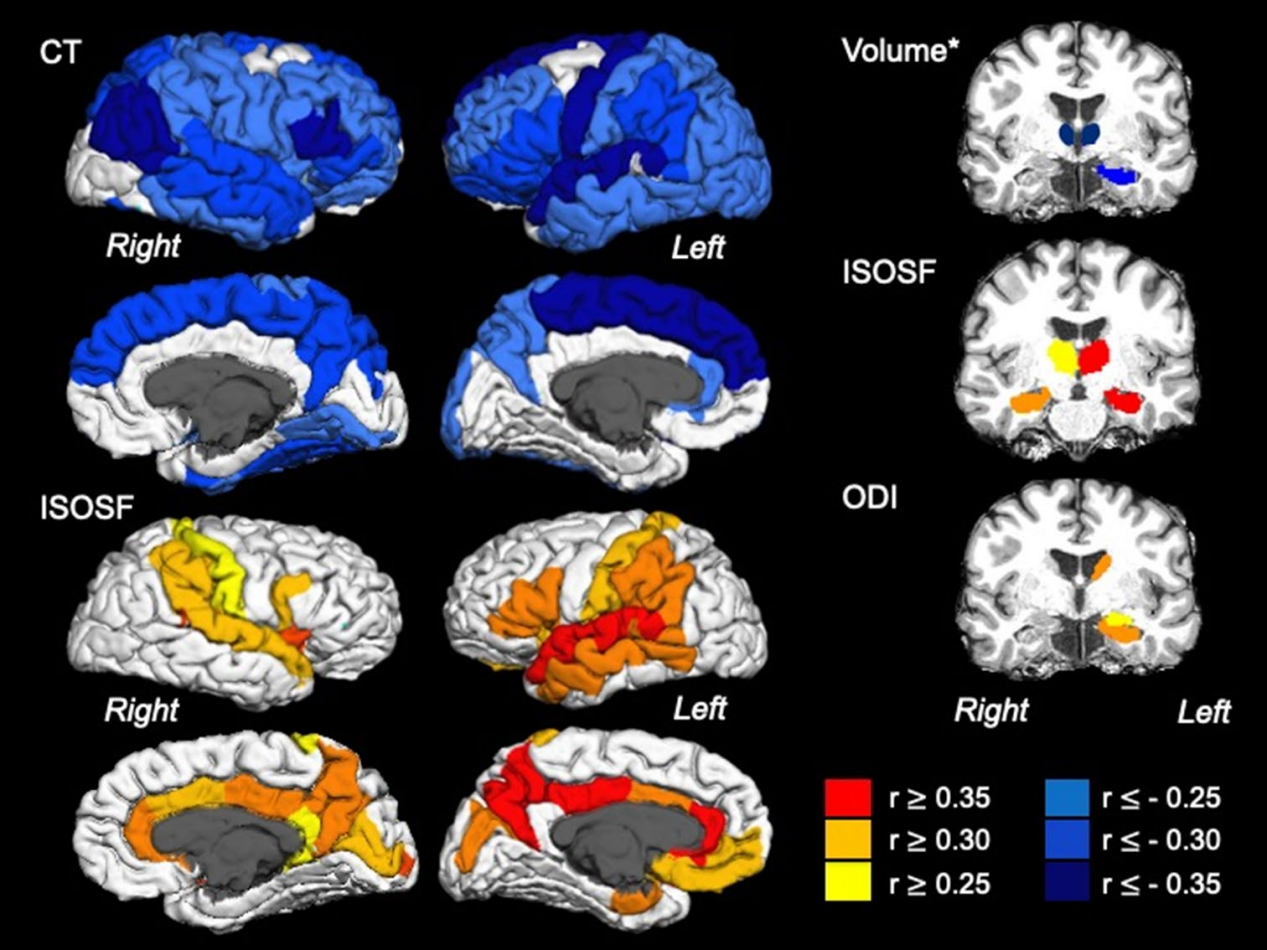
displays the effects of age on cortical thickness (CT), subcortical volume (corrected for intracranial volume), isotropic signal fraction (ISOSF) and orientation dispersion index (ODI) across 34 cortical regions per hemisphere parcellated with the Desikan–Killiany atlas^121^ and seven subcortical regions per hemisphere (hippocampus, amygdala, thalamus, caudate, putamen, globus pallidus, nucleus accumbens). Region of interest segmentations were performed with FreeSurfer (version 5.3). Regions are colour-coded according to the size of the age effect indicated by Pearson correlation coefficient r. Warm colours indicate positive and blue colours negative correlations.

Ageing was associated with bilateral increases in ISOSF in medial regions including the cingulate, precuneus and cuneus cortices and in lateral regions including superior temporal, supramarginal, postcentral, pars opercularis and insula cortices. Age-related increases in ISOSF were also observed in left middle temporal and pars triangularis regions as well as in subcortical hippocampi, thalami, nuclei accumbens and right putamen (Table 4) (Fig. 3). Men relative to women had higher ISOSF in widespread frontal, temporal, parietal and cingulate cortices and in caudate nuclei, hippocampi, thalami and right nucleus accumbens (Table 4) (Fig. 2). In addition, NART-IQ correlated positively with ISOSF in the superior temporal sulci (left: r = 0.253, p_BHadj_ = 0.008; right: r = 0.241, p_BHadj_ = 0.006), left superior parietal (r = 0.227, p_BHadj_ = 0.006), and right lingual (r = 0.182, p_BHadj_ = 0.026) cortices (Table 4). After partialling out of age only correlations on the left hemisphere remained significant [superior parietal cortex [(r = 0.206, p_BHadj_ = 0.048), superior temporal sulcus (r = 0.197, p_BHadj_ = 0.032)] but those on the right did not [superior temporal sulcus (p_BHadj_ = 0.053), lingual (p_BHadj_ = 0.08)].

**Table 4:** Post-hoc effects of *age,* sex and NART-IQ on the isotropic signal fraction (ISOSF).

Effect	Side	ROI	F_(1,119)_-value	p_BHadj_
Age	Left	**Accumbens**	**16.946**	** < 0.001**
		Amygdala	0.002	0.977
		Caudate	2.906	0.174
		**Hippocampus**	**32.296**	** < 0.001**
		Pallidum	0.741	0.544
		Putamen	3.705	0.121
		**Thalamus**	**17.881**	** < 0.001**
	Right	**Accumbens**	**8.272**	**0.016**
		Amygdala	0.090	0.847
		Caudate	4.359	0.090
		**Hippocampus**	**20.305**	** < 0.001**
		Pallidum	0.168	0.787
		**Putamen**	**6.089**	**0.039**
		**Thalamus**	**21.716**	** < 0.001**
	Left	**Banks of superior temporal sulcus**	**12.121**	**0.003**
		**Caudal anterior cingulate**	**12.152**	**0.004**
		**Cuneus**	**17.203**	** < 0.001**
		Entorhinal	0.170	0.788
		Frontal pole	0.667	0.559
		Fusiform	0.884	0.494
		**Inferior parietal**	**6.381**	**0.035**
		Inferior temporal	0.765	0.538
		**Insula**	**17.457**	** < 0.001**
		**Lateral occipital**	**6.671**	**0.031**
		Lateral orbito frontal	3.029	0.163
		Lingual	2.481	0.212
		**Medial orbito frontal**	**6.335**	**0.035**
		**Middle temporal**	**11.334**	**0.004**
		Paracentral	4.216	0.095
		Parahippocampal	0.125	0.819
		**Pars opercularis**	**19.568**	** < 0.001**
		Pars orbitalis	0.005	0.961
		**Pars triangularis**	**15.445**	** < 0.001**
		**Postcentral**	**14.471**	** < 0.001**
		**Posterior cingulate**	**15.798**	** < 0.001**
		Precentral	5.314	0.057
		**Precuneus**	**19.354**	** < 0.001**
		**Rostral anterior cingulate**	**16.241**	** < 0.001**
		Rostral middle frontal	5.017	0.067
		Superior frontal	1.173	0.410
		Superior parietal	0.963	0.470
		**Superior temporal**	**25.891**	** < 0.001**
		**Supramarginal**	**16.621**	** < 0.001**
		Temporal pole	1.219	0.410
		**Transverse temporal**	**51.576**	** < 0.001**
	Right	**Banks of superior temporal sulcus**	**12.346**	**0.003**
		**Caudal anterior cingulate**	**7.267**	**0.025**
		**Cuneus**	**13.388**	** < 0.001**
		Entorhinal	0.131	0.819
		Frontal pole	1.185	0.414
		Fusiform	0.108	0.835
		Inferior parietal	1.881	0.297
		Inferior temporal	1.475	0.366
		**Insula**	**14.803**	** < 0.001**
		**Isthmus cingulate**	**6.659**	**0.031**
		Lateral occipital	1.818	0.307
		Lateral orbito frontal	1.286	0.406
		**Lingual**	**7.195**	**0.024**
		Medial orbito frontal	3.288	0.147
		Middle temporal	3.039	0.165
		Paracentral	0.702	0.556
		PARAHIPPOCAMPAL	1.158	0.412
		**Pars opercularis**	**15.415**	** < 0.001**
		Pars orbitalis	2.665	0.195
		Pars triangularis	0.523	0.605
		**Pericalcerine**	**16.505**	** < 0.001**
		Postcentral	6.318	0.034
		**Posterior cingulate**	**18.89**	** < 0.001**
		Precentral	4.015	0.104
		**Precuneus**	**15.968**	** < 0.001**
		**Rostral anterior cingulate**	**12.476**	**0.003**
		Rostral middle frontal	2.466	0.212
		Superior frontal	0.676	0.550
		Superior parietal	3.634	0.124
		**Superior temporal**	**12.296**	**0.003**
		**Supramarginal**	**8.563**	**0.013**
		Temporal pole	2.727	0.189
		**Transverse temporal**	**44.346**	** < 0.001**
Sex	Left	Accumbens	4.687	0.078
		Amygdala	0.320	0.693
		**Caudate**	**6.885**	**0.029**
		**Hippocampus**	**30.457**	** < 0.001**
		Pallidum	3.735	0.120
		Putamen	0.886	0.497
		**Thalamus**	**6.685**	**0.031**
	Right	**Accumbens**	**10.982**	**0.003**
		Amygdala	3.110	0.161
		**Caudate**	**8.610**	**0.013**
		**Hippocampus**	**37.739**	** < 0.001**
		Pallidum	1.177	0.412
		Putamen	0.595	0.577
		**Thalamus**	**28.188**	** < 0.001**
	Left	**Banks of superior temporal sulcus**	**9.745**	**0.007**
		**Caudal anterior cingulate**	**10.321**	**0.007**
		**Cuneus**	**14.189**	** < 0.001**
		Entorhinal	2.097	0.263
		Frontal pole	1.317	0.400
		Fusiform	0.471	0.621
		**Inferior parietal**	**19.193**	** < 0.001**
		Inferior temporal	3.546	0.129
		**Insula**	**14.093**	** < 0.001**
		**Lateral occipital**	**15.940**	** < 0.001**
		Lateral orbito frontal	0.039	0.902
		Lingual	1.178	0.414
		Medial orbito frontal	3.411	0.138
		**Middle temporal**	**17.995**	** < 0.001**
		Paracentral	1.542	0.355
		**Parahippocampal**	**14.537**	** < 0.001**
		**Pars opercularis**	**11.519**	**0.003**
		Pars orbitalis	0.167	0.784
		**Pars triangularis**	**16.204**	** < 0.001**
		**Postcentral**	**28.162**	** < 0.001**
		**Posterior cingulate**	**16.237**	** < 0.001**
		**Precentral**	**22.987**	** < 0.001**
		**Precuneus**	**13.571**	** < 0.001**
		Rostral anterior cingulate	4.385	0.088
		**Rostral middle frontal**	**35.530**	** < 0.001**
		**Superior frontal**	**13.064**	** < 0.001**
		**Superior parietal**	**18.143**	** < 0.001**
		**Superior temporal**	**26.621**	** < 0.001**
		**Supramarginal**	**42.479**	** < 0.001**
		Temporal pole	4.436	0.088
		Transverse temporal	30.601	< 0.001
	Right	**Banks of superior temporal sulcus**	**14.697**	** < 0.001**
		**Caudal anterior cingulate**	**10.623**	**0.004**
		**Cuneus**	**24.330**	** < 0.001**
		Entorhinal	0.491	0.616
		Frontal pole	0.684	0.557
		Fusiform	3.168	0.158
		**Inferior parietal**	**6.885**	**0.030**
		Inferior temporal	3.105	0.162
		Insula	4.265	0.094
		Isthmus cingulate	0.601	0.578
		**Lateral occipital**	**10.275**	**0.006**
		Lateral orbito frontal	0.102	0.839
		**Lingual**	**7.981**	**0.019**
		Medial orbito frontal	3.038	0.166
		Middle temporal	5.352	0.055
		**Paracentral**	**9.075**	**0.010**
		Parahippocampal	3.733	0.121
		**Pars opercularis**	**7.161**	**0.027**
		Pars orbitalis	3.870	0.112
		**Pars triangularis**	**5.958**	**0.042**
		**Pericalcerine**	**14.080**	** < 0.001**
		**Postcentral**	**19.109**	** < 0.001**
		**Posterior cingulate**	**14.954**	** < 0.001**
		**Precentral**	**17.777**	** < 0.001**
		**Precuneus**	**13.291**	** < 0.001**
		**Rostral anterior cingulate**	**5.785**	**0.046**
		**Rostral middle frontal**	**24.380**	** < 0.001**
		**Superior frontal**	**16.120**	** < 0.001**
		**Superior parietal**	**8.266**	**0.016**
		**Superior temporal**	**16.902**	** < 0.001**
		**Supramarginal**	**16.983**	** < 0.001**
		Temporal pole	0.330	0.691
		**Transverse temporal**	**37.792**	** < 0.001**
NART-IQ	Left	Accumbens	0.709	0.556
		Amygdala	3.741	0.120
		Caudate	0.016	0.932
		Hippocampus	0.065	0.864
		Pallidum	0.022	0.922
		Putamen	1.221	0.411
		Thalamus	0.000	0.995
	Right	Accumbens	0.022	0.924
		Amygdala	1.266	0.410
		Caudate	1.809	0.306
		Hippocampus	0.067	0.866
		Pallidum	0.206	0.764
		Putamen	0.606	0.579
		Thalamus	0.481	0.618
	Left	**Banks of superior temporal sulcus**	**6.816**	**0.029**
		Caudal anterior cingulate	0.035	0.901
		Cuneus	0.200	0.767
		Entorhinal	0.343	0.684
		Frontal pole	1.745	0.315
		Fusiform	0.039	0.904
		Inferior parietal	2.029	0.274
		Inferior temporal	0.019	0.925
		Insula	4.834	0.073
		Lateral occipital	0.306	0.697
		Lateral orbito frontal	0.037	0.901
		Lingual	0.621	0.574
		Medial orbito frontal	0.000	0.993
		Middle temporal	0.402	0.655
		Paracentral	0.199	0.764
		Parahippocampal	0.010	0.943
		Pars opercularis	0.207	0.768
		Pars orbitalis	1.006	0.459
		Pars triangularis	0.636	0.570
		Postcentral	1.370	0.388
		Posterior cingulate	1.243	0.411
		Precentral	0.401	0.653
		Precuneus	0.078	0.852
		Rostral anterior cingulate	0.582	0.581
		Rostral middle frontal	1.208	0.411
		Superior frontal	1.224	0.414
		**Superior parietal**	**6.435**	**0.033**
		Superior temporal	0.266	0.724
		Supramarginal	0.879	0.493
		Temporal pole	0.084	0.849
		Transverse temporal	2.832	0.180
	Right	**Banks of superior temporal sulcus**	**6.815**	**0.030**
		Caudal anterior cingulate	0.530	0.605
		Cuneus	2.829	0.179
		Entorhinal	4.702	0.077
		Frontal pole	1.644	0.332
		Fusiform	2.222	0.246
		Inferior parietal	2.952	0.170
		Inferior temporal	0.001	0.987
		Insula	0.090	0.843
		Isthmus cingulate	1.257	0.409
		Lateral occipital	0.126	0.821
		Lateral orbito frontal	0.014	0.933
		**Lingual**	**5.866**	**0.044**
		Medial orbito frontal	0.318	0.692
		Middle temporal	0.097	0.842
		Paracentral	2.527	0.208
		Parahippocampal	1.983	0.280
		Pars opercularis	0.242	0.741
		Pars orbitalis	0.050	0.888
		Pars triangularis	0.502	0.613
		Pericalcerine	2.623	0.198
		Postcentral	1.806	0.306
		Posterior cingulate	1.662	0.331
		Precentral	0.685	0.559
		Precuneus	2.629	0.197
		Rostral anterior cingulate	0.453	0.628
		Rostral middle frontal	0.394	0.653
		Superior frontal	1.525	0.355
		Superior parietal	4.186	0.096
		Superior temporal	0.002	0.978
		Supramarginal	1.407	0.381
		Temporal pole	4.445	0.087
		Transverse temporal	0.024	0.923

p_BHadj_, 5% False Discovery Rate Benjamini–Hochberg adjusted *p* value; ROI, Region of Interest. Significant results are highlighted in bold.

#### ODI omnibus effects

There was a significant main effect of age [F(78,51) = 2.0, p_BHadj_ = 0.040, η_p_^2^ = 0.8] and a significant interaction effect between FH and WHR [F(78,51) = 2.3, p_BHadj_ = 0.010, η_p_^2^ = 0.8] but no main effects for sex (p_BHadj_ = 0.270), NART-IQ (p_BHadj_ = 0.497), *APOE* (p_BHadj_ = 0.153), FH (p_BHadj_ = 0.520) or WHR (p_BHadj_ = 0.330) and no interaction effects between *APOE* and FH (p_BHadj_ = 0.436), *APOE* and WHR (p_BHadj_ = 0.295) or *APOE*, FH and WHR (p_BHadj_ = 0.228) were observed.

#### ODI post-hoc effects

Age-related increases in ODI were observed in left hippocampus, amygdala, caudate and right transverse temporal cortex (Table 5) (Fig. 3).

**Table 5:** Post-hoc effects of *age* on the orientation dispersion index (ODI).

Effect	Side	ROI	F_(1,128)_-value	p_BHadj_
Age	Left	Accumbens	3.529	0.307
		**Amygdala**	**16.646**	** < 0.001**
		**Caudate**	**13.995**	** < 0.001**
		**Hippocampus**	**15.638**	** < 0.001**
		Pallidum	0.017	0.958
		Putamen	3.880	0.306
		Thalamus	2.111	0.505
	Right	Accumbens	1.265	0.594
		Amygdala	7.018	0.156
		Caudate	0.040	0.925
		Hippocampus	8.834	0.124
		Pallidum	0.365	0.755
		Putamen	2.142	0.506
		Thalamus	0.148	0.828
	Left	Banks of superior temporal sulcus	2.793	0.398
		Caudal anterior cingulate	7.199	0.156
		Cuneus	0.001	0.992
		Entorhinal	5.518	0.222
		Frontal pole	2.182	0.515
		Fusiform	2.889	0.387
		Inferior parietal	0.029	0.943
		Inferior temporal	1.654	0.559
		Insula	0.579	0.698
		Lateral occipital	1.619	0.563
		Lateral orbito frontal	1.572	0.560
		Lingual	0.919	0.616
		Medial orbito frontal	5.107	0.253
		Middle temporal	1.088	0.598
		Paracentral	0.634	0.693
		Parahippocampal	0.173	0.826
		Pars opercularis	0.076	0.892
		Pars orbitalis	2.068	0.507
		Pars triangularis	0.055	0.914
		Postcentral	0.526	0.705
		Posterior cingulate	1.419	0.575
		Precentral	0.305	0.776
		Precuneus	0.063	0.907
		Rostral anterior cingulate	1.459	0.576
		Rostral middle frontal	2.006	0.496
		Superior frontal	1.109	0.595
		Superior parietal	4.078	0.326
		Superior temporal	2.666	0.409
		Supramarginal	0.291	0.760
		Temporal pole	8.362	0.130
		Transverse temporal	0.200	0.817
	Right	Banks of superior temporal sulcus	0.534	0.712
		Caudal anterior cingulate	2.715	0.408
		Cuneus	0.628	0.691
		Entorhinal	1.911	0.516
		Frontal pole	3.977	0.312
		Fusiform	2.329	0.479
		Inferior parietal	0.004	0.984
		Inferior temporal	4.430	0.288
		Insula	4.760	0.268
		Isthmus cingulate	5.750	0.216
		Lateral occipital	1.311	0.591
		Lateral orbito frontal	1.274	0.598
		Lingual	0.173	0.819
		Medial orbito frontal	0.734	0.666
		Middle temporal	4.509	0.295
		Paracentral	0.899	0.611
		Parahippocampal	0.373	0.754
		Pars opercularis	2.490	0.445
		Pars orbitalis	1.778	0.544
		Pars triangularis	0.023	0.952
		Pericalcerine	0.293	0.765
		Postcentral	1.564	0.553
		Posterior cingulate	0.042	0.926
		Precentral	0.100	0.870
		Precuneus	0.000	0.985
		Rostral anterior cingulate	0.284	0.760
		Rostral middle frontal	0.268	0.768
		Superior frontal	0.485	0.716
		Superior parietal	3.130	0.352
		Superior temporal	5.045	0.238
		Supramarginal	1.426	0.581
		Temporal pole	6.156	0.198
		**Transverse temporal**	**10.589**	**0.039**

p_BHadj_, 5% False Discovery Rate Benjamini–Hochberg adjusted *p* value; *ROI* region of interest. Significant results are highlighted in bold.

Post-hoc effects for the interaction between FH and WHR did not survive 5% FDR correction (Supplementary Table 2).

#### ICSF effects

There were no significant main or interaction effects on ICSF [age (p_BHadj_ = 0.170), sex (p_BHadj_ = 0.130), NART-IQ (p_BHadj_ = 0.451), *APOE* (p_BHadj_ = 0.324), FH (p_BHadj_ = 0.342), WHR (p_BHadj_ = 0.517), *APOE* × FH (p_BHadj_ = 0.541), *APOE* × WHR(p_BHadj_ = 0.236) , FH × WHR (p_BHadj_ = 0.883), *APOE* × FH × WHR (p_BHadj_ = 0.912)].

### MANCOVA on cortical thickness and subcortical volume (ICV corrected)

#### Omnibus effects

There were main effects for age [F(82,68) = 1.8, p_BHadj_ = 0.035, η_p_^2^ = 0.7] and sex [F(82,68) = 1.9, p_BHadj_ = 0.040, η_p_^2^ = 0.7]. No main effects were observed for *APOE* (p_BHadj_ = 0.597), FH (p_BHadj_ = 0.144), WHR (p_BHadj_ = 0.152) or NART-IQ (p_BHadj_ = 0.651). No interaction effects between *APOE* and FH (p_BHadj_ = 0.844), *APOE* and WHR (p_BHadj_ = 0.978), FH and WHR (p_BHadj_ = 0.053) or *APOE*, FH and WHR (p_BHadj_ = 0.123) were observed.

#### Post-hoc effects

Ageing was associated with widespread thinning in bilateral frontal, temporal, and parietal cortical regions as well as with volume loss in subcortical structures, i.e. in the left hippocampus, left nucleus accumbens, bilateral thalami and putamen (Table 6) (Fig. 3). Women relative to men had larger volumes in left hippocampus, left nucleus accumbens, left putamen, right caudate and right pallidum. They also had larger cortical thickness in the right isthmus cingulate but lower cortical thickness in the left insula (Table 6) (Fig. 2).

**Table 6:** Post-hoc effects of age and sex on cortical thickness and subcortical volume measures.

Effect	Side	ROI	Index	F_(1,149)_-value	p_BHadj_
Age	Left	**Accumbens**	**Vol**_**ICVadj**_	**7.037**	**0.027**
		Amygdala	Vol_ICVadj_	3.360	0.146
		Caudate	Vol_ICVadj_	0.073	0.873
		**Hippocampus**	**Vol**_**ICVadj**_	**12.023**	**0.004**
		Pallidum	Vol_ICVadj_	1.141	0.448
		**Putamen**	**Vol**_**ICVadj**_	**8.886**	**0.012**
		**Thalamus**	**Vol**_**ICVadj**_	**26.144**	** < 0.001**
	Right	Accumbens	Vol_ICVadj_	4.944	0.071
		Amygdala	Vol_ICVadj_	3.723	0.120
		Caudate	Vol_ICVadj_	0.225	0.778
		Hippocampus	Vol_ICVadj_	2.828	0.190
		Pallidum	Vol_ICVadj_	2.444	0.221
		**Putamen**	**Vol**_**ICVadj**_	**7.722**	**0.021**
		**Thalamus**	**Vol**_**ICVadj**_	**45.557**	** < 0.001**
	Left	**Banks of superior temporal sulcus**	**CT**	**5.798**	**0.047**
		Caudal anterior cingulate	CT	0.583	0.589
		**Caudal middle frontal**	**CT**	**8.485**	**0.016**
		Cuneus	CT	3.911	0.110
		Entorhinal	CT	0.120	0.836
		Frontal pole	CT	0.076	0.885
		Fusiform	CT	5.474	0.057
		**Inferior parietal**	**CT**	**11.874**	**0.004**
		**Inferior temporal**	**CT**	**7.261**	**0.027**
		**Insula**	**CT**	**20.522**	** < 0.001**
		Isthmus cingulate	CT	0.130	0.836
		Lateral occipital	CT	4.536	0.086
		**Lateral orbito frontal**	**CT**	**12.478**	**0.006**
		**Lingual**	**CT**	**6.891**	**0.030**
		**Medial orbito frontal**	**CT**	**7.171**	**0.026**
		**Middle temporal**	**CT**	**12.759**	** < 0.001**
		**Paracentral**	**CT**	**20.354**	** < 0.001**
		**Parahippocampal**	**CT**	**7.647**	**0.022**
		**Pars opercularis**	**CT**	**14.469**	** < 0.001**
		**Pars orbitalis**	**CT**	**18.893**	** < 0.001**
		**Pars triangularis**	**CT**	**19.089**	** < 0.001**
		Pericalcerine	CT	2.678	0.203
		**Postcentral**	**CT**	**12.426**	**0.006**
		Posterior cingulate	CT	1.032	0.467
		**Precentral**	**CT**	**28.246**	** < 0.001**
		**Precuneus**	**CT**	**12.353**	**0.006**
		**Rostral anterior cingulate**	**CT**	**7.759**	**0.022**
		**Rostral middle frontal**	**CT**	**13.280**	** < 0.001**
		**Superior frontal**	**CT**	**24.962**	** < 0.001**
		**Superior parietal**	**CT**	**9.821**	**0.009**
		**Superior temporal**	**CT**	**27.155**	** < 0.001**
		**Supramarginal**	**CT**	**22.159**	** < 0.001**
		Temporal pole	CT	0.682	0.555
		Transverse temporal	CT	2.574	0.211
	Right	**Banks of superior temporal sulcus**	**CT**	**11.955**	**0.006**
		Caudal anterior cingulate	CT	3.192	0.150
		Caudal middle frontal	CT	2.576	0.209
		Cuneus	CT	1.553	0.363
		Entorhinal	CT	0.121	0.840
		Frontal pole	CT	0.015	0.938
		**Fusiform**	**CT**	**18.048**	** < 0.001**
		**Inferior parietal**	**CT**	**22.640**	** < 0.001**
		**Inferior temporal**	**CT**	**9.714**	**0.008**
		**Insula**	**CT**	**12.353**	**0.005**
		Isthmus cingulate	CT	4.464	0.088
		Lateral occipital	CT	4.184	0.099
		**Lateral orbito frontal**	**CT**	**13.295**	** < 0.001**
		**Lingual**	**CT**	**7.316**	**0.026**
		**Medial orbito frontal**	**CT**	**6.738**	**0.029**
		**Middle temporal**	**CT**	**18.517**	** < 0.001**
		**Paracentral**	**CT**	**17.110**	** < 0.001**
		**Parahippocampal**	**CT**	**8.659**	**0.015**
		**Pars opercularis**	**CT**	**12.395**	**0.005**
		**Pars orbitalis**	**CT**	**12.59**	**0.005**
		**Pars triangularis**	**CT**	**19.087**	** < 0.001**
		Pericalcerine	CT	2.454	0.221
		**Postcentral**	**CT**	**7.200**	**0.025**
		**Posterior cingulate**	**CT**	**6.381**	**0.038**
		**Precentral**	**CT**	**10.001**	**0.009**
		**Precuneus**	**CT**	**15.729**	** < 0.001**
		Rostral anterior cingulate	CT	1.949	0.290
		**Rostral middle frontal**	**CT**	**10.641**	**0.005**
		**Superior frontal**	**CT**	**18.426**	** < 0.001**
		**Superior parietal**	**CT**	**7.745**	**0.021**
		**Superior temporal**	**CT**	**19.439**	** < 0.001**
		**Supramarginal**	**CT**	**10.607**	**0.005**
		Temporal pole	CT	0.020	0.950
		Transverse temporal	CT	1.548	0.359
Sex	Left	**Accumbens**	**Vol**_**ICVadj**_	**8.927**	**0.012**
		Amygdala	Vol_ICVadj_	0.074	0.878
		Caudate	Vol_ICVadj_	4.492	0.086
		**Hippocampus**	**Vol**_**ICVadj**_	**10.913**	**0.007**
		Pallidum	Vol_ICVadj_	1.649	0.343
		**Putamen**	**Vol**_**ICVadj**_	**6.103**	**0.042**
		Thalamus	Vol_ICVadj_	1.934	0.289
	Right	Accumbens	Vol_ICVadj_	3.833	0.113
		Amygdala	Vol_ICVadj_	0.513	0.623
		**Caudate**	**Vol**_**ICVadj**_	**7.183**	**0.025**
		Hippocampus	Vol_ICVadj_	4.695	0.080
		**Pallidum**	**Vol**_**ICVadj**_	**7.633**	**0.020**
		Putamen	Vol_ICVadj_	4.265	0.096
		Thalamus	Vol_ICVadj_	4.360	0.090
	Left	Banks of superior temporal sulcus	CT	3.183	0.157
		Caudal anterior cingulate	CT	0.019	0.935
		Caudal middle frontal	CT	0.018	0.934
		Cuneus	CT	1.857	0.302
		Entorhinal	CT	0.075	0.881
		Frontal pole	CT	0.794	0.519
		Fusiform	CT	0.285	0.761
		Inferior parietal	CT	2.104	0.268
		Inferior temporal	CT	0.229	0.780
		**Insula**	**CT**	**9.485**	**0.008**
		Isthmus cingulate	CT	0.031	0.928
		Lateral occipital	CT	0.244	0.772
		Lateral orbito frontal	CT	0.058	0.886
		Lingual	CT	0.891	0.503
		Medial orbito frontal	CT	1.146	0.455
		Middle temporal	CT	0.206	0.783
		Paracentral	CT	2.266	0.244
		Parahippocampal	CT	0.936	0.490
		Pars opercularis	CT	1.245	0.436
		Pars orbitalis	CT	0.134	0.837
		Pars triangularis	CT	2.647	0.204
		Pericalcerine	CT	0.202	0.782
		Postcentral	CT	4.122	0.100
		Posterior cingulate	CT	0.295	0.759
		Precentral	CT	0.008	0.948
		Precuneus	CT	0.098	0.859
		Rostral anterior cingulate	CT	0.038	0.917
		Rostral middle frontal	CT	0.019	0.941
		Superior frontal	CT	1.171	0.451
		Superior parietal	CT	0.459	0.649
		Superior temporal	CT	0.141	0.835
		Supramarginal	CT	4.028	0.105
		Temporal pole	CT	1.133	0.447
		Transverse temporal	CT	1.466	0.377
	Right	Banks of superior temporal sulcus	CT	3.084	0.166
		Caudal anterior cingulate	CT	0.069	0.872
		Caudal middle frontal	CT	0.809	0.527
		Cuneus	CT	0.855	0.513
		Entorhinal	CT	0.746	0.536
		Frontal pole	CT	1.243	0.433
		Fusiform	CT	0.799	0.522
		Inferior parietal	CT	5.173	0.063
		Inferior temporal	CT	0.019	0.946
		Insula	CT	5.346	0.059
		**Isthmus cingulate**	**CT**	**6.254**	**0.037**
		Lateral occipital	CT	0.625	0.574
		Lateral orbito frontal	CT	2.769	0.193
		Lingual	CT	0.267	0.770
		Medial orbito frontal	CT	0.941	0.493
		Middle temporal	CT	0.167	0.811
		Paracentral	CT	2.089	0.267
		Parahippocampal	CT	1.127	0.444
		Pars opercularis	CT	0.993	0.478
		Pars orbitalis	CT	0.670	0.556
		Pars triangularis	CT	0.007	0.944
		Pericalcerine	CT	0.008	0.959
		Postcentral	CT	2.954	0.178
		Posterior cingulate	CT	0.704	0.550
		Precentral	CT	0.252	0.771
		Precuneus	CT	0.806	0.524
		Rostral anterior cingulate	CT	1.115	0.444
		Rostral middle frontal	CT	0.008	0.953
		Superior frontal	CT	0.003	0.959
		Superior parietal	CT	4.903	0.072
		Superior temporal	CT	0.220	0.777
		Supramarginal	CT	1.145	0.451
		Temporal pole	CT	0.005	0.951
		Transverse temporal	CT	0.262	0.768

*CT* cortical thickness; *Vol*_*ICVadj*_ volume adjusted for intracranial volume. p_BHadj_, 5% False Discovery Rate Benjamini–Hochberg adjusted *p* value; *ROI* region of interest.

### Exploring interaction effects between APOE, age and sex

Potential interaction effects between *APOE*, age and sex on left thalamus MPF were explored. Univariate analysis of variance revealed an effect of *APOE* [F(1,141) = 5.7, *p* = 0.018] and age [F(2,141) = 3.7, *p* = 0.027] but no interaction effects between *APOE* and age (*p* = 0.700) or *APOE* and sex (*p* = 0.900).

### Exploring moderator effects of blood pressure and markers of inflammation

We then explored with two separate analyses of covariances whether controlling for differences in (i) systolic and diastolic blood pressure (BP) and (ii) inflammation-related measures of C-Reactive Protein (CRP), Interleukin-8 (IL-8) and leptin/adiponectin ratio (LAR) would account for the effect of *APOE* on left thalamus MPF.

While no covariate showed a main effect [systolic BP (*p* = 0.680), diastolic BP (*p* = 0.750), CRP (*p* = 0.150), IL-8 (*p* = 0.400), LAR (*p* = 0.500)], the *APOE* effect on the left thalamus MPF remained significant [F(1,149) = 6.7, p_BHadj_ = 0.030] after accounting for BP measures, but was not significant anymore after controlling for CRP, IL-8 and LAR (*p* = 0.060).

## Discussion

Here, we investigated whether qMRI indices of apparent neurite density and dispersion, free water, myelin, and cell metabolism were sensitive to grey matter differences related to LOAD risk in cognitively healthy individuals. Such microstructural measurements hold the potential for novel imaging biomarkers to identify asymptomatic individuals at heightened risk of developing LOAD. As such they may provide non-invasive and cheaper alternatives to PET and cerebrospinal fluid (CSF)-based biomarkers, that are currently employed in clinical trials, in the future.

The only significant difference between asymptomatic *APOE*-ε4 carriers relative to non-carriers was in the qMT measure MPF in the left thalamus with *APOE*-ε4 related reductions in MPF (Fig. 1). This effect was observed independently of age, sex, and verbal intelligence. Reduced MPF may arise from processes that lead to an increase in free water and/or a reduction in the macromolecular content of grey matter including changes in myelin, proteins, and and/or iron concentrations^68,69^. Such changes may be consistent with the presence of inflammatory processes leading to tissue swelling associated with glia activation^70^ and/or with a deficit in cholesterol transport in *APOE*-ε4 carriers ^70–72^. Consistent with this interpretation we observed that the effect of *APOE* genotype on left thalamus MPF was moderated by plasma markers of inflammation (CRP, IL-8, LAR). Furthermore, evidence suggests that *APOE*-ε4 carriage may increase susceptibility to inflammation^22,23^ and that inflammatory processes contribute significantly to the pathogenesis of LOAD^73–75^.

Notably these *APOE*-ε4-related differences in MPF were only observed in the left thalamus but not in any other cortical or subcortical region. The limbic thalamic nuclei maintain dense reciprocal connections with the hippocampal formation and the retrosplenial cortex^76,77^, which, together with the fornix, mamillary bodies and posterior cingulate cortex, comprise the Papez circuit important for episodic memory function^78^. As outlined above it is increasingly recognised that the Papez circuit, including the anterior thalamus, can be affected early in LOAD^4^. Neurofibrillary accumulations are found in the anterodorsal thalamic nucleus at the same time as those in the hippocampus in LOAD brains^34^ and neuroimaging studies have revealed reduced thalamic volume in both amnestic MCI^35^ and LOAD^36^. Furthermore, studies into the effects of *APOE* in middle-aged asymptomatic adults found reduced glucose metabolism in the thalamus, hippocampus and cingulate cortex^39^ as well as increased metabolism in bilateral thalami and superior temporal gyrus in amyloid-β positive *APOE*-ε4 carriers with a maternal history of LOAD^79^. Cacciaglia et al.^80^ studied the effects of *APOE* on grey matter volume in over 500 middle-aged asymptomatic individuals and identified reduced hippocampus, caudate, precentral gyrus, and cerebellum volumes but increased volumes in the thalamus, superior frontal and middle occipital gyri in *APOE*-ε4 carriers. While it remains unknown why *APOE*-ε4 may be related to increased thalamic volume it was suggested that this could reflect brain swelling associated with glial activation in response to larger amyloid-β burden^81^. As mentioned above, the here observed pattern of *APOE*-ε4-related reductions in MPF in the left thalamus is consistent with this interpretation^56,82^. One other study investigated the impact of *APOE*-ε4 on qMT white matter metrics in young adults and did not find any differences^83^. This suggests that such risk-related glial dysfunction may accumulate with age and may only become apparent from midlife onwards.

The question arises why we did not observe any risk-related effects in brain regions that have previously been reported to be affected by LOAD risk factors^10,84,85^. Reports with regards to the impact of *APOE*-ε4 on grey matter structures in healthy young and middle-aged adults have been mixed^10,84^, with some studies reporting no changes in hippocampal grey matter volume in *APOE*-ε4 carriers^31,86^. Studies assessing the impact of *APOE*-ε4 on tissue microstructure have primarily focused on diffusion tensor imaging (DTI) of white matter. While some reported widespread white matter differences in DTI measures^83,87,88^, this has not been replicated in all studies^30,89^. These discrepancies may arise due to DTI indices not being sufficiently sensitive and/or specific to detect early risk-related tissue abnormalities^90^. Direct comparisons between DTI and NODDI indices revealed that although fractional anisotropy (FA) was sensitive to white matter differences between healthy controls and patients with metabolic disease, FA was less anatomically specific and did not identify all brain regions that were captured by ICSF and ODI^91^. Thus we employed NODDI and qMT measurements to study risk effects on grey matter here and on white matter in a previous CARDS analysis^92^. In the previously published white matter analysis^92^ we did not observe any main effects of risk but found that individuals with the highest genetic risk (obese FH + and *APOE*-ε4) exhibited obesity-related reductions in MPF and ICSF in the right parahippocampal cingulum.

Taken together, our previous and here reported findings demonstrate that MPF from qMT can identify risk-related microstructural differences in limbic grey and white matter that were not apparent in conventional volumetric or cortical thickness measurements. We propose that these differences may reflect subtle changes related to neuroglia activation and that limbic structures including the thalamus are particularly susceptible to adverse effects of *APOE*-ε4 on glia cells. Inconsistencies in previous studies may have arisen from standard morphological and DTI measurements not being sensitive and/or specific enough to detect such glia-related changes.

It is important to note that while we did not find any risk-related effects on brain morphology we did replicate the well-established pattern of widespread age-related thinning in frontal, temporal and parietal regions^93^ as well as volume loss in subcortical structures including the hippocampi and thalami (Fig. 3). The subcortical volume loss was accompanied by age-related increases in ISOSF in bilateral hippocampi and thalami but effects on cortical regions were more localised: increased ISOSF was apparent along medial regions of the cingulate and parietal cortices including the precuneus as well as in superior temporal and lateral and orbito prefrontal cortices. Age-related increases in ISOSF have been previously observed^94^ and most likely reflect lost tissue being replaced by CSF. Consistent with a previous study^95^ we also observed a positive correlation between age and ODI, an estimate of neurite dispersion, in the hippocampus and the left caudate and amygdala. In contrast to Nazari et al.^95^ however, we did not find any effects in cortical regions, while they reported reduced ODI with age in fronto-parietal regions. These opposing patterns in cortical and subcortical regions may reflect age-related reductions of neocortical dendritic spine density^96^ with accompanying compensatory increases in the dendritic extent of dentate gyrus granular cells^97,98^. Similar age-related increases in the dendritic tree have also been reported in the basolateral nucleus of the amygdala of rats^99^.

Furthermore, we observed positive correlations between ISOSF and NART-IQ in superior temporal, parietal and lingual cortices that were partly driven by age. NART requires the reading of irregularly pronounced words and older relative to younger adults tended to perform better in the NART. However, positive albeit weak correlations between NART-IQ and ISOSF remained for the left superior temporal sulcus and left superior parietal cortex. Developmental imaging studies have revealed cortical thinning during adolescence^100^ that may be due to increased myelination^100^ or synaptic pruning and dendritic arborization^101,102^. It may therefore be possible that childhood developmental differences in cortical maturation as well as in education may have contributed to this effect. For instance, childhood cognitive abilities have been found to account for relationships between cognitive performance and brain cortical thickness decades later in older adults from the Lothian birth cohort^103^.

Consistent with previous reports^104^ we did not observe widespread sex-differences in brain morphology measurements with the exception of larger volumes in the left hippocampus in women than men^105^. However, qMRI indices revealed the following pattern: Women compared to men, had lower ISOSF in widespread cortical and subcortical regions and larger MPF in frontal and temporal regions. Previously we also reported higher MPF and lower ISOSF for white matter in women than men^44^. Overall this pattern of sex differences suggests higher cortical myelination and lower free water signal in women as they tended to be overall in better health i.e. were less obese, had lower systolic BP, and reported drinking less alcohol than men^44^. All of these factors may have contributed to women showing “healthier” grey and white matter in the CARDS cohort.

Finally, some study limitations need to be considered. First of all, CARDS is a cross-sectional study that cannot answer whether the observed *APOE* effects on left thalamus MPF are predictive of accelerated development of LOAD pathology, cognitive, or neuronal decline. Future prospective longitudinal studies are required to address this question. We also propose that our findings require replication in larger samples that can control for possible interactions between *APOE* and other LOAD risk genes such as variants of *TREM*2 and polygenic risk hazards as the number of participants in the CARDS study was too small to do so. It is also worth mentioning that other qMRI measurements, that were not included in the current study, may prove helpful in characterising risk effects on the brain. Notably quantitative T_2_ and T_2_^*^ measurements have been proposed to be sensitive to neurodegenerative processes. For instance, prolonged T_2_ relaxometry has been reported in the hippocampus of LOAD patients^106^ and has been proposed to increase the sensitivity and specificity of MCI and LOAD detection^107^. Finally, it should be noted that we only studied the thalamus as a whole structure while neuropathological evidence suggests a specific vulnerability of the anterodorsal thalamic nucleus to LOAD pathology. Future studies may investigate risk-related effects on specific subthalamic nuclei, which was beyond the scope of the current study as we were focusing on risk effects across the whole brain.

In summary, we have shown *APOE*-ε4 related reductions in the qMT measure MPF in the left thalamus that were moderated by peripheral markers of inflammation. This effect occurred independently of age, sex and NART-IQ and was not observed in morphological or microstructural indices from diffusion-weighted imaging. In addition, the effect was specific to the left thalamus and was not present in other cortical and subcortical grey matter regions. We propose that MPF reductions may reflect the effects of glia-mediated inflammatory and demyelination processes in *APOE*-ε4 carriers. As such qMT measurements hold the potential for non-invasive and cheaper biomarker alternatives to PET, that may aid our understanding of the pathological processes leading to LOAD. In addition, qMT may help with the identification of asymptomatic individuals at heightened risk of LOAD for stratification into clinical trials for future preventative therapeutics.

## Materials and methods

The Cardiff Ageing and Risk of Dementia Study (CARDS) has been described previously including a detailed description of the participant sample^43,92^, assessment of genetic and metabolic risk factors^44,92^ and the acquisition and processing of the MRI data^43,44,92,108^. Here we provide a brief summary of the most important points. CARDS received ethical approval from the School of Psychology Research Ethics Committee at Cardiff University (EC.14.09.09.3843R2) and all participants provided written informed consent in accordance with the Declaration of Helsinki. All research methods were performed in line with Cardiff University’s Research Integrity and Governance Code of Practice and relevant data protection regulations.

### Participants

The CARDS cohort comprised 166 community-dwelling individuals between the age of 38 and 71 years who underwent cognitive and health assessment as well as MRI scanning (Table 1). Exclusion criteria were a history of neurological and/or psychiatric disease, head injury, drug/alcohol dependency, high risk cardio-embolic source, large-vessel disease or MRI incompatibility due to pacemaker, stents or other surgical implants. As a group, participants intellectual functioning was above average as assessed with the National Adult Reading Test (NART)^66^. All but one participant scored > 26 on the Mini Mental State Exam (MMSE)^42^ thus the remaining 165 participants were classified as cognitively healthy. Eight participants scored ≥ 10 in the Patient Health Questionnaire (PHQ)-9^109^, suggesting moderate levels of depression but no participant was severely depressed.

### Assessment of risk factors

Saliva samples were collected with the Genotek Oragene-DNA kit (OG-500) and *APOE* genotypes ε2, ε3, and ε4 were determined with TaqMan genotyping of single nucleotide polymorphism (SNP) rs7412 and KASP genotyping of SNP rs429358. Participants self-reported their family history of dementia, i.e., whether a first-grade relative was affected by Alzheimer’s disease, vascular dementia or any other type of dementia.

Central obesity was assessed from the waist-hip ratio (WHR)^44^ with abdominal obesity defined as a WHR ≥ 0.9 for males and ≥ 0.85 for females. Resting systolic and diastolic blood pressure (BP) readings were taken with a digital blood pressure monitor (Model UA-631; A&D Medical, Tokyo, Japan) and the means of three readings were calculated. Participants self-reported other metabolic risk factors, including diabetes mellitus, high levels of blood cholesterol controlled with statin medication, history of smoking, and weekly alcohol intake. There were only few diabetics, smokers, and individuals on statins and, hence, these variables were not included in the analyses.

### Blood plasma analysis

As previously reported^44,92^, venous blood samples were drawn into 9 ml heparin coated plasma tubes after 12 h overnight fasting and were centrifuged for 10 min at 2000 × *g* within 1 h from blood collection. Plasma samples were then transferred into 0.5 ml polypropylene microtubes and stored in a freezer at − 80 °C. Circulating levels of high-sensitivity C-Reactive Protein (CRP) in mg/dL were assayed using a human CRP Quantikine enzyme-linked immunosorbent assay (ELISA) kit (R & D Systems, Minneapolis, USA). Six individuals had a CRP value > 10 mg/ml indicative of acute infection and were, therefore, excluded from the statistical analyses testing for moderating effects of inflammation. Leptin concentrations in pg/ml were determined with the DRP300 Quantikine ELISA kit (R & D Systems) and adiponectin in ng/ml with the human total adiponectin/Acrp30 Quantitkine ELISA kit (R & D Systems). Leptin/adiponectin ratios for each participant were calculated. Interleukin IL-8 levels in pg/mL were determined using a high sensitivity CXCL8/ INTERLEUKIN-8 Quantikine ELISA kit (R & D Systems). Determination of interleukin-1β, interleukin-6 and Tumor Necrosis Factor α (TNFα) levels were trialled with high-sensitivity Quantikine ELISA kits but did not result in reliable measurements consistently above the level of detection for each assay.

### MRI data acquisition

MRI data were acquired on a 3 T MAGNETOM Prisma clinical scanner (Siemens Healthcare, Erlangen, Germany) as described in^43,44,92,108^. T_1_-weighted images (1 × 1 × 1 mm voxel) were collected with a three-dimension (3D) magnetization-prepared rapid gradient-echo (MP-RAGE) sequence (256 × 256 acquisition matrix, TR = 2300 ms, TE = 3.06 ms, TI = 850 ms, flip angle θ = 9°, 176 slices, 1 mm slice thickness, FOV = 256 mm and acquisition time of ~ 6 min).

High Angular Resolution Diffusion Imaging (HARDI)^51^ data (2 × 2 × 2 mm voxel) were collected with a spin-echo echo-planar dual shell HARDI sequence with diffusion encoded along 90 isotropically distributed orientations^110^ (30 directions at b-value = 1200 s/mm^2^ and 60 directions at b-value = 2400 s/mm^2^) and six non-diffusion weighted scans with dynamic field correction and the following parameters: TR = 9400 ms, TE = 67 ms, 80 slices, 2 mm slice thickness, FOV = 256 × 256 × 160 mm, GRAPPA acceleration factor = 2 and acquisition time of ~ 15 min.

Quantitative magnetization transfer weighted imaging (qMT) data were acquired with a prototype sequence, i.e. an optimized 3D MT-weighted gradient-recalled-echo sequence^46^ to obtain magnetization transfer-weighted data with the following parameters: TR = 32 ms, TE = 2.46 ms; Gaussian MT pulses, duration t = 12.8 ms; FA = 5°; FOV = 24 cm, 2.5 × 2.5 × 2.5 mm^3^ resolution. The following off-resonance irradiation frequencies (Θ) and their corresponding saturation pulse nominal flip angles (ΔSAT) for the 11 MT-weighted images were optimized using Cramer-Rao lower bound optimization: Θ = [1000 Hz, 1000 Hz, 2750 Hz, 2768 Hz, 2790 Hz, 2890 Hz, 1000 Hz, 1000 Hz, 12,060 Hz, 47,180 Hz, 56,360 Hz] and their corresponding ΔSAT values = [332°, 333°, 628°, 628°, 628°, 628°, 628°, 628°, 628°, 628°, 332°]. The longitudinal relaxation time, T_1_, of the system was estimated by acquiring three 3D gradient recalled echo sequence (GRE) volumes with three different flip angles (θ = 3°,7°,15°) using the same acquisition parameters as used in the MT-weighted sequence (TR = 32 ms, TE = 2.46 ms, FOV = 24 cm, 2.5 × 2.5 × 2.5 mm^3^ resolution). Data for computing the static magnetic field (B_0_) were collected using two 3D GRE volumes with different echo-times (TE = 4.92 ms and 7.38 ms respectively; TR = 330 ms; FOV = 240 mm; slice thickness 2.5 mm)^111^. The acquisition time for the complete qMT sequence including all fieldmaps was ~ 30 min.

### HARDI and qMT data processing

As described in^43,44,92,108^, the dual-shell HARDI data were split and b = 1200 and 2400 s/mm^2^ data were corrected separately for distortions induced by the diffusion-weighted gradients and motion artifacts with appropriate reorientation of the encoding vectors^112^ in ExploreDTI (Version 4.8.3)^113^. EPI-induced geometrical distortions were corrected by warping the diffusion-weighted image volumes to the T_1_—weighted anatomical images^114^. After pre-processing, the NODDI model^45^ was fitted to the HARDI data with the fast, linear model fitting algorithms of the Accelerated Microstructure Imaging via Convex Optimization (AMICO) framework^115^ to gain ISOSF, ICSF, and ODI maps.

Using Elastix^116^, MT-weighted GRE volumes were co-registered to the MT-volume with the most contrast using a rigid body (6 degrees of freedom) registration to correct for inter-scan motion. Data from the 11 MT-weighted GRE images and T_1_-maps were fitted by a two-pool model using the Ramani pulsed-MT approximation^117^. This approximation provided MPF and *k*_*f*_ maps. To remove voxels with noise-only data, MPF maps were thresholded to an upper intensity limit of 0.3 and *k*_*f*_ maps to an upper limit of 3.0 using the fslmaths imaging calculator from the Functional Magnetic Resonance Imaging of the Brain (FMRIB) library (version 6).

All image modality maps were spatially aligned to the T_1_-weighted anatomical volume as reference image with linear affine registration (12 degrees of freedom) in within-subject space using FMRIB’s Linear Image Registration Tool (FLIRT)^118,119^.

### Cortical and subcortical grey matter region segmentation

Grey matter cortical and subcortical regions were automatically segmented from T_1_—weighted images with the Freesurfer image analysis suite (version 5.3), which is documented online (https://surfer.nmr.mgh.harvard.edu/)^64^. The images were processed by running the “recon-all” script using the default analysis settings. In brief, the images were registered to the Montreal Neurological Institute standard space and intensity normalization was performed. This was followed by automatic skull stripping to remove extracerebral structures, the cerebellum and the brain stem, followed by segmentation into grey matter, white matter and CSF and separation of the hemispheres. Pial surfaces were obtained by tessellating the grey and white matter boundary and by surface deformation following intensity gradients for optimal placement of grey and white matter and grey matter and CSF boundaries^120^. Surface inflation and registration to a spherical atlas were then performed and the cerebral cortex was parcellated into 34 regions per hemisphere based on gyral and sulcal structures following the Desikan-Killiany atlas^121^. Cortical thickness measurements were estimated as the average shortest distance between the pial surface and the white matter boundary^122^. For each hemisphere, seven deep grey matter structures (hippocampus, amygdala, thalamus, caudate, putamen, pallidum, and nucleus accumbens) were automatically parcellated using a probabilistic atlas so that average volumetric measurements could be determined^123,124^. Mean intracranial volume fractions (ICV) were extracted for each brain as estimates of individual differences in head sizes and all volumetric measurements were adjusted for ICV by dividing each participant’s subcortical volume by their ICV.

Finally, the mean values of all microstructural indices were extracted from each participants’ cortical and subcortical region of interests. Mean measurements were taken in each participants’ native space. This was done by first converting each participants’ cortical and subcortical masks from the FreeSurfer Massachusetts General Hospital volume file format (MGZ) into the Neuroimaging Informations Technology Initiative (NIfTI) analyze-style data format and then uploading the microstructural maps onto each region of interest mask using the fslmaths command from the FMRIB library. Mean values of each index for each mask were then extracted using the FMRIB fslstats command. NODDI and qMT indices of ISOSF, ICSF, ODI, MPF and *k*_*f*_, could not be extracted from bilateral caudal middle frontal, left isthmus cingulate and left pericalcarine regions and R_1_ could not be extracted from the right postcentral region.

## Supplementary information

Supplementary Information.
