# Supplementary material for: APOE-ε4-related differences in left thalamic microstructure in cognitively healthy adults
Source: Sci Rep. 2020 Nov 13;10:19787. doi: 10.1038/s41598-020-75992-9 (PMC7666117; doi:10.1038/s41598-020-75992-9)
Supplement: Supplementary file 1 — Supplementary Information. [file 41598_2020_75992_MOESM1_ESM.docx]

**Title**: *APOE*-ε4-related differences in left thalamic microstructure in cognitively healthy adults

**Abbreviated title** *APOE*-ε4 effects on thalamic microstructure

**Authors:** Jilu P. Mole^1^, Fabrizio Fasano^2^, John Evans^1^, Rebecca Sims^3^, Emma Kidd^4^, John P. Aggleton^1^ & Claudia Metzler-Baddeley^1&^

^&^Corresponding author email: [Metzler-BaddeleyC@cardiff.ac.uk](mailto:Metzler-BaddeleyC@cardiff.ac.uk)

**Affiliation**s: ^1^Cardiff University Brain Research Imaging Centre (CUBRIC), School of Psychology, Cardiff University, Maindy Road, Cathays, Cardiff, CF24 4HQ; ^2^Siemens Healthcare, Henkestrasse 127, 91052 Erlangen, Germany; ^3^Division of Psychological Medicine and Clinical Neuroscience, School of Medicine, Cardiff University, Haydn Ellis Building, Maindy Road, Cathays, Cardiff, CF24 4HQ. ^4^School of Pharmacy and Pharmaceutical Sciences, Cardiff University, Redwood Building, King Edward VII Avenue, Cardiff, CF10 3NB.

Supplementary Table 1. Post-hoc comparisons for *APOE* genotype on R_1_.

| **Effect** | **Side** | **ROI** | **F_(1,124)_-value** | **p_BHadj_** |
| --- | --- | --- | --- | --- |
| *APOE* | left | accumbens | 1.977 | 0.830 |
|  |  | amygdala | 1.623 | 0.840 |
|  |  | caudate | 0.472 | 0.986 |
|  |  | hippocampus | 0.143 | 1.000 |
|  |  | pallidum | 0.773 | 1.000 |
|  |  | putamen | 0.088 | 1.000 |
|  |  | thalamus | 0.093 | 1.000 |
|  | right | accumbens | 0.023 | 0.987 |
|  |  | amygdala | 0.135 | 1.000 |
|  |  | caudate | 2.985 | 0.891 |
|  |  | hippocampus | 0.011 | 0.964 |
|  |  | pallidum | 0.413 | 0.949 |
|  |  | putamen | 0.598 | 1.000 |
|  |  | thalamus | 0.181 | 1.000 |
|  | left | banks of superior temporal sulcus | 2.881 | 0.838 |
|  |  | caudal anterior cingulate | 1.327 | 0.857 |
|  |  | caudal middle frontal | 1.263 | 0.862 |
|  |  | cuneus | 0.772 | 1.000 |
|  |  | entorhinal | 0.013 | 0.979 |
|  |  | frontal pole | 2.285 | 0.991 |
|  |  | fusiform | 0.452 | 0.935 |
|  |  | inferior parietal | 0.839 | 1.000 |
|  |  | inferior temporal | 0.679 | 1.000 |
|  |  | insula | 6.534 | 0.492 |
|  |  | isthmus cingulate | 0.002 | 1.000 |
|  |  | lateral occipital | 0.228 | 1.000 |
|  |  | lateral orbito frontal | 0.261 | 1.000 |
|  |  | lingual | 0.460 | 0.951 |
|  |  | medial orbito frontal | 0.000 | 1.000 |
|  |  | middle temporal | 2.145 | 0.798 |
|  |  | paracentral | 0.026 | 0.993 |
|  |  | parahippocampal | 0.089 | 1.000 |
|  |  | pars opercularis | 7.485 | 0.574 |
|  |  | pars orbitalis | 0.042 | 1.000 |
|  |  | pars triangularis | 0.016 | 0.996 |
|  |  | pericalcerine | 0.547 | 0.994 |
|  |  | postcentral | 2.277 | 0.915 |
|  |  | posterior cingulate | 0.030 | 0.996 |
|  |  | precentral | 2.867 | 0.762 |
|  |  | precuneus | 0.000 | 1.000 |
|  |  | rostral anterior cingulate | 0.516 | 0.971 |
|  |  | rostral middle frontal | 0.106 | 1.000 |
|  |  | superior frontal | 0.469 | 0.966 |
|  |  | superior parietal | 0.182 | 1.000 |
|  |  | superior temporal | 0.698 | 1.000 |
|  |  | supramarginal | 2.220 | 0.876 |
|  |  | temporal pole | 3.399 | 0.929 |
|  |  | transverse temporal | 5.222 | 0.492 |
|  | right | banks of superior temporal sulcus | 0.047 | 1.000 |
|  |  | caudal anterior cingulate | 6.164 | 0.382 |
|  |  | caudal middle frontal | 0.013 | 0.968 |
|  |  | cuneus | 0.112 | 1.000 |
|  |  | entorhinal | 0.056 | 1.000 |
|  |  | frontal pole | 0.035 | 1.000 |
|  |  | fusiform | 1.849 | 0.848 |
|  |  | inferior parietal | 0.833 | 1.000 |
|  |  | inferior temporal | 0.261 | 1.000 |
|  |  | insula | 3.741 | 0.902 |
|  |  | isthmus cingulate | 0.942 | 1.000 |
|  |  | lateral occipital | 0.047 | 1.000 |
|  |  | lateral orbito frontal | 0.567 | 1.000 |
|  |  | lingual | 3.080 | 0.960 |
|  |  | medial orbito frontal | 1.676 | 0.854 |
|  |  | middle temporal | 0.250 | 1.000 |
|  |  | paracentral | 0.033 | 1.000 |
|  |  | parahippocampal | 2.177 | 0.837 |
|  |  | pars opercularis | 1.577 | 0.827 |
|  |  | pars orbitalis | 0.652 | 1.000 |
|  |  | pars triangularis | 0.000 | 1.000 |
|  |  | pericalcerine | 0.546 | 0.969 |
|  |  | posterior cingulate | 1.126 | 0.917 |
|  |  | precentral | 0.303 | 1.000 |
|  |  | precuneus | 0.146 | 1.000 |
|  |  | rostral anterior cingulate | 1.727 | 0.870 |
|  |  | rostral middle frontal | 0.180 | 1.000 |
|  |  | superior frontal | 0.016 | 0.984 |
|  |  | superior parietal | 1.412 | 0.883 |
|  |  | superior temporal | 1.393 | 0.855 |
|  |  | supramarginal | 0.090 | 1.000 |
|  |  | temporal pole | 0.606 | 1.000 |
|  |  | transverse temporal | 0.038 | 1.000 |

No results were significant after multiple comparison correction. p_BHadj_, 5% False Discovery Rate Benjamini-Hochberg adjusted p-value; ROI, Region of Interest.

Supplementary Table 2. Post-hoc comparisons for the omnibus interaction effect between Family History and Waist-Hip-Ration on the orientation dispersion index (ODI).

| **Effect** | **Side** | **ROI** | **F_(1,128)_-value** | **p_BHadj_** |
| --- | --- | --- | --- | --- |
| *FH x WHR* | left | accumbens | 0.346 | 0.755 |
|  |  | amygdala | 0.823 | 0.641 |
|  |  | caudate | 1.159 | 0.590 |
|  |  | hippocampus | 0.144 | 0.820 |
|  |  | pallidum | 1.211 | 0.583 |
|  |  | putamen | 0.009 | 0.969 |
|  |  | thalamus | 0.701 | 0.677 |
|  | right | accumbens | 0.492 | 0.719 |
|  |  | amygdala | 0.078 | 0.895 |
|  |  | caudate | 0.164 | 0.823 |
|  |  | hippocampus | 0.002 | 0.984 |
|  |  | pallidum | 0.045 | 0.927 |
|  |  | putamen | 1.770 | 0.537 |
|  |  | thalamus | 5.556 | 0.208 |
|  | left | banks of superior temporal sulcus | 1.226 | 0.585 |
|  |  | caudal anterior cingulate | 0.350 | 0.759 |
|  |  | cuneus | 3.818 | 0.295 |
|  |  | entorhinal | 0.959 | 0.618 |
|  |  | frontal pole | 2.926 | 0.390 |
|  |  | fusiform | 1.514 | 0.565 |
|  |  | inferior parietal | 0.022 | 0.948 |
|  |  | inferior temporal | 0.919 | 0.609 |
|  |  | insula | 2.152 | 0.514 |
|  |  | lateral occipital | 3.834 | 0.300 |
|  |  | lateral orbito frontal | 0.002 | 0.987 |
|  |  | lingual | 3.525 | 0.297 |
|  |  | medial orbito frontal | 1.013 | 0.608 |
|  |  | middle temporal | 1.001 | 0.606 |
|  |  | paracentral | 0.397 | 0.758 |
|  |  | parahippocampal | 1.750 | 0.533 |
|  |  | pars opercularis | 0.147 | 0.823 |
|  |  | pars orbitalis | 0.007 | 0.969 |
|  |  | pars triangularis | 1.408 | 0.571 |
|  |  | postcentral | 0.300 | 0.773 |
|  |  | posterior cingulate | 0.616 | 0.690 |
|  |  | precentral | 0.297 | 0.769 |
|  |  | precuneus | 1.893 | 0.513 |
|  |  | rostral anterior cingulate | 3.774 | 0.290 |
|  |  | rostral middle frontal | 0.548 | 0.712 |
|  |  | superior frontal | 0.756 | 0.669 |
|  |  | superior parietal | 0.184 | 0.821 |
|  |  | superior temporal | 4.439 | 0.274 |
|  |  | supramarginal | 0.531 | 0.708 |
|  |  | temporal pole | 2.039 | 0.507 |
|  |  | transverse temporal | 0.452 | 0.726 |
|  | right | banks of superior temporal sulcus | 0.661 | 0.693 |
|  |  | caudal anterior cingulate | 1.129 | 0.595 |
|  |  | cuneus | 3.699 | 0.296 |
|  |  | entorhinal | 0.934 | 0.616 |
|  |  | frontal pole | 0.000 | 0.990 |
|  |  | fusiform | 3.158 | 0.357 |
|  |  | inferior parietal | 3.936 | 0.305 |
|  |  | inferior temporal | 0.608 | 0.688 |
|  |  | insula | 2.014 | 0.503 |
|  |  | isthmus cingulate | 0.954 | 0.614 |
|  |  | lateral occipital | 1.328 | 0.593 |
|  |  | lateral orbito frontal | 0.012 | 0.963 |
|  |  | lingual | 1.604 | 0.559 |
|  |  | medial orbito frontal | 0.310 | 0.777 |
|  |  | middle temporal | 0.395 | 0.753 |
|  |  | paracentral | 4.064 | 0.312 |
|  |  | parahippocampal | 1.235 | 0.591 |
|  |  | pars opercularis | 0.196 | 0.815 |
|  |  | pars orbitalis | 0.224 | 0.801 |
|  |  | pars triangularis | 6.772 | 0.156 |
|  |  | pericalcerine | 3.582 | 0.306 |
|  |  | postcentral | 0.014 | 0.961 |
|  |  | posterior cingulate | 1.071 | 0.598 |
|  |  | precentral | 1.245 | 0.595 |
|  |  | precuneus | 7.550 | 0.156 |
|  |  | rostral anterior cingulate | 6.225 | 0.182 |
|  |  | rostral middle frontal | 0.381 | 0.756 |
|  |  | superior frontal | 1.176 | 0.590 |
|  |  | superior parietal | 0.650 | 0.692 |
|  |  | superior temporal | 0.750 | 0.665 |
|  |  | supramarginal | 1.037 | 0.604 |
|  |  | temporal pole | 0.470 | 0.720 |
|  |  | transverse temporal | 0.160 | 0.821 |

No results were significant after multiple comparison correction. p_BHadj_, 5% False Discovery Rate Benjamini-Hochberg adjusted p-value; ROI, Region of Interest.
